# Nonalcoholic fatty liver disease, a potential risk factor of non-specific ST-T segment changes: data from a cross-sectional study

**DOI:** 10.7717/peerj.9090

**Published:** 2020-05-13

**Authors:** Li Xiao, Tao Bai, Junchao Zeng, Rui Yang, Ling Yang

**Affiliations:** 1 Division of Gastroenterology, Union Hospital, Tongji Medical College, Huazhong University of Science and Technology, Wuhan, China; 2 Physical Examination (Health Management) Center, Union Hospital, Tongji Medical College, Huazhong University of Science and Technology, Wuhan, China

**Keywords:** Nonalcoholic fatty liver disease, Non-specific ST-T segment, Risk factor, Odds ratio, Confidence interval

## Abstract

**Background:**

Non-specific ST-T segment changes are prevalent and are proven risk factors for early onset of cardiovascular diseases. They can increase all-cause mortality by 100∼200% and are candidate for early signs of cardiovascular changes. Nonalcoholic fatty liver disease (NAFLD) is prevalent worldwide and is one facet of a multisystem disease that confers substantial increases morbidity and mortality of nonalcoholic fatty liver-related cardiovascular diseases. It is unclear whether NAFLD is associated with non-specific ST-T changes warning early signs of cardiovascular changes. Therefore, we investigated this association.

**Methods:**

A cross-sectional study was designed that included a sample consisting of 32,922 participants who underwent health examinations. Participants with missing information, excessive alcohol intake, viral hepatitis, chronic liver disease or established cardiovascular diseases were excluded. Electrocardiograms were used for analysis of non-specific ST-T segment changes. NAFLD was diagnosed by ultrasonographic detection of hepatic steatosis without other liver diseases. A multivariable logistic regression model was served to calculate the OR and 95% CI for non-specific ST-T segment changes.

**Results:**

The prevalence of non-specific ST-T segment changes was 6.5% in participants with NAFLD, however, the prevalence of NAFLD was 42.9% in participants with non-specific ST-T segment changes. NAFLD was independently associated with non-specific ST-T segment changes (OR: 1.925, 95% CI: 1.727-2.143, *P* < 0.001). After adjusting for age, sex, heart rate, hypertension, body mass index, fasting glucose, total cholesterol, triglycerides, HDL-C, NAFLD remained an independent risk factor of non-specific ST-T segment changes (OR: 1.289, 95% CI: 1.122-1.480).

**Conclusion:**

Non-specific ST-T segment changes were independently associated with the presence of NAFLD after adjusting for potential confounders.

## Introduction

Nonalcoholic fatty liver disease (NAFLD) has emerged as the most frequent chronic liver disease worldwide (Patel & Siddiqui, 2019), ranging from simple steatosis to steatohepatitis, cirrhosis, and hepatocellular cancer (Fuchs, Claudel & Trauner, 2014; Targher, Day & Bonora, 2010). Meanwhile, cardiovascular disease (CVD) is the most common cause of death, and its prevalence is constantly increasing worldwide (Balakumar, Maung & Jagadeesh, 2016). Many studies have shown that NAFLD increases not only the risk of morbidity and mortality of liver-related cardiovascular disease, but also independently increases the risk for multi-vessel CVD associated with cardiovascular events (Anstee, Targher & Day, 2013; Boddi et al., 2013; Chang et al., 2019; Targher, Day & Bonora, 2010). Furthermore, a large body of literature currently demonstrates the association between NAFLD and cardiac arrhythmias; in particular, NAFLD is associated with an increased risk of atrial fibrillation, QT interval prolongation and ventricular tachyarrhythmia (Hung et al., 2015; Mantovani et al., 2016; Targher et al., 2013; Targher et al., 2014). Therefore, it is urgent to early detect nonalcoholic fatty liver-related cardiovascular diseases.

Non-specific ST-T segment (NST) changes are characterized by a slightly upswept ST-segment depression or downward sloping and flat, diphasic or inverted T waves on a resting 12-lead electrocardiogram (ECG) (Kumar et al., 2008). In clinical practice, such abnormalities are frequently encountered but generally ignored or simply considered to be incidental, transient, and benign electrocardiographic changes (Greenland et al., 2003; Kumar & Lloyd-Jones, 2007). In addition, diverse morphological ECG abnormalities complicate the reasonable explanation of NST changes (Rautaharju et al., 2009). Physicians previously treated NST alterations as unremarkable changes. However, studies have shown that the all-cause mortality is increased by two to three times in patients with NST changes (Badheka et al., 2012). Furthermore, minor abnormalities have been reported to be independent risk factors for early CVD events and incident coronary heart disease (CHD) in middle-aged and older individuals, and the degree of risk increase is similar to that of elevated levels of traditional CVD risk factors, such as diabetes mellitus, hypercholesterolemia, and hypertension (Greenland et al., 2003; Healy & Lloyd-Jones, 2016; Kumar et al., 2008; Rivero et al., 2019; Walsh et al., 2013). The existing evidence suggests that NST changes may be overlooked early signs of cardiovascular changes.

NAFLD is one facet of multisystem metabolic abnormalities. Previous studies have indicated that NAFLD could indicate an early stage of change during the process of CVD. Because it is important to predict and identify of the onset of CVD earlier, we aimed to evaluate the association between NAFLD and NST changes, hoping for greater vigilance for early prevention of CVD events, especially, in patients with NAFLD.

## Methods

### Participants

**Figure 1:**
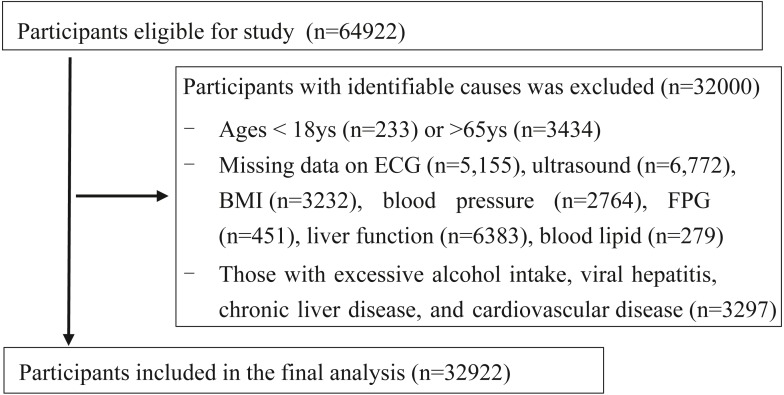
Flowchart of participants selection. ECG, Electrocardiogram; FPG, fasting plasma glucose; BMI, body mass index.

A total of 64,922 recordings were retrospectively reviewed at the physical examination centre in Wuhan Union Hospital from January 2017 to December 2018. All the participants were company staff or workers who came for annual health examination. A total of 68.5% participants from the local companies in Wuhan, 18% from the other cities outside of Wuhan in Hubei province and 14.5% from other provinces. For the current cross-sectional study, we excluded 32,000 subjects for the following reasons: (1) aged <18 years or >65 years (*n* = 3667), (2) missing information on ECG, ultrasonography, or important covariates including body mass index (BMI), blood pressure (BP), fasting plasma glucose (FPG), liver function, and blood lipids (*n* = 25,036), (3) patients with excessive alcohol intake, viral hepatitis, chronic liver disease, or CVD (*n* = 3297). Some participants met more than one exclusion criterion, leaving 32,922 participants included in the final analysis (Fig. 1). Tongji Medical College, Huazhong University of Science and Technology granted Ethical approval to carry out the study within its facilities (2018 S426). This study was for retrospective analysis only, so informed consent was waived.

### Clinical and laboratory data

The BMI was calculated as weight divided by height squared (kg/m^2^). Blood pressure was recorded with a mercury sphygmomanometer in a sitting position. Participants were considered to be hypertensive if their systolic blood pressure was ≥ 140 mm Hg or/and their diastolic blood pressure was ≥ 90 mm Hg. Fasting blood samples were drawn from the antecubital vein. Blood tests included fasting plasma glucose (FPG), total cholesterol (TC), triglycerides (TG), high-density lipoprotein (HDL-C), and low-density lipoprotein (LDL-C), alamine aminotransferase (ALT), aspartate aminotransferase (AST), alkaline phosphatase (ALP), gamma-glutamyl transferase (GGT).

### Hepatic ultrasonography

Hepatic ultrasonic examinations were performed in all participants by experienced ultrasonographers who were blinded to the clinical and laboratory data. Hepatic steatosis was determined based on standard criteria, including the presence of a diffuse increase in echogenicity compared to the cortex of the ipsilateral kidney, posterior attenuation of the ultrasound beam, and poor visualization of the walls of the portal vein (Chang et al., 2019). The severity of hepatic steatosis was also recorded as no (less than 5%), mild (≥5–33%), or moderate (>33–66%) and severe (≥66%) on sonography. Because the number of severe steatosis was too small, the steatosis of NAFLD was classified as absent, mild and moderate/severe.

### ECG and non-specific ST-T segment changes

A standard 12-lead electrocardiogram (ECG) was recorded for detection of NST changes, which were specifically defined according to Minnesota Codes 4-3, 4-4, 5-3, and 5-4 (Prineas, Crow & Zhang, 2010).

### Statistical analysis

Data are expressed as the mean ± standard deviation (SD), median (interquartile range) or proportion. An unpaired *t*-test, one-way ANOVA and the chi-squared test were used to compare differences among the clinical characteristics of the participants categorized by NST changes. Four regression models were performed to progressively reduce the impact of potential confounders on the association between NAFLD and NST changes. Basic model 1 was unadjusted; multivariable model 2 was adjusted for age and sex; model 3 included the heart rate, presence of hypertension (blood pressure ≥ 140/90 mmHg) and fasting plasma glucose plus the same variables included in model 2, and finally, the analysis was further adjusted for the same variables included in model 3 plus BMI, TC, TG, and HDL-C (model 4). Covariates included in the multivariate regression models were chosen as potential confounders based on their biological plausibility or statistical association with NST changes in univariable analyses.

All statistical analyses were performed with SPSS 21.0, and all *P* values were two-tailed. Values of *P* <0.05 were considered statistically significant.

## Results

### Characteristics of participants

The clinical and biochemical characteristics of participants stratified by NST changes were summarized in Table 1. NAFLD was present in 9454 (28.7%) patients who underwent health examinations. The prevalence of non-specific ST-T segment changes was 6.5% in participants with NAFLD, however, the prevalence of NAFLD was 42.9% in participants with non-specific ST-T segment changes. The mean ages (SD) of patients with and without NST changes were 50 years and 41 years (*P* <0.001), respectively, a higher prevalence presented in middle-aged and older individuals. The mean BMI (SD) was 23.4 kg/m^2^ in patients without NST changes and 24.7 kg/m^2^ in patients with NST changes, suggesting that overweight people may be more likely to have NST changes. Patients with NST changes tended to be female (60.7% vs 39.3% in males, *P* <0.001). In patients with NST changes, the incidence of mild steatosis and moderate/severe steatosis were 35.3% and 7.6%, respectively. In addition, NST changes were associated with blood pressure, and higher levels of fasting plasma glucose, triglycerides, total cholesterol, LDL-C, and lower levels of HDL-C.

**Table 1:** Clinical characteristics of participants with electrocardiogram and non-specific ST-T segment changes.

	Normal	Non-specific ST-T segment changes	Chi-square	*P*-value
	n (%) or median (quartile)	n (%) or median (quartile)		
Sex			108.506	<0.001
Female (n)	14701 (46.7)	872 (60.7)		
Male (n)	16785 (53.3)	564 (39.3)		
Age (years)	41.0 (32.0–49.0)	50.0 (42.0–57.0)	−26.345	<0.001
Height (cm)	167.0 (160.0–171.5)	163.5 (157.5–170.5)	−11.856	<0.001
Weight (kg)	64.5 (56.0–73.8)	65.4 (57.2–75.5)	−3.862	<0.001
BMI (kg/m^2^)	23.4 (21.2–25.7)	24.7 (22.4–27.1)	−13.733	<0.001
Heart rate (bpm)	74.0 (69.0–80.0)	74.0 (69.0–81.0)	−0.304	0.761
Systolic blood pressure (mmHg)	117.0 (106.0–128.0)	125.0 (110.0–140.0)	−18.430	<0.001
Diastolic blood pressure (mmHg)	75.0 (70.0–80.0)	80.0 (74.0–90.0)	−20.054	<0.001
Fasting plasma glucose (mmol/l)	4.9 (4.6–5.3)	5.1 (4.8–5.7)	−12.591	<0.001
Triglyceride (mmol/l)	1.2 (0.8–1.8)	1.4 (1.0–2.1)	−10.355	<0.001
AST (U/L)	21.0 (17.0–26.0)	22.0 (18.0–28.0)	−6.610	<0.001
ALT (U/L)	21.0 (15.0–32.0)	23.0 (16.0–35.0)	−4.651	<0.001
ALP (U/L)	68.0 (57.0–82.0)	73.0 (59.0–89.0)	−8.351	<0.001
GGT (U/L)	20.0 (14.0–33.0)	22.0 (15.3–38.0)	−5.967	<0.001
Total cholesterol (mmol/l)	4.8 (4.2–5.3)	5.1 (4.5–5.7)	−12.130	<0.001
HDL-C (mmol/l)	1.4 (1.3–1.6)	1.4 (1.3–1.7)	−2.848	0.004
LDL-C (mmol/l)	2.5 (2.1–3.1)	2.7 (2.2–3.3)	−8.615	<0.001
NAFLD severity on ultrasound (%)			148.115	<0.001
No steatosis	22648 (71.9)	820 (57.1)		
Mild steatosis	7181 (22.8)	507 (35.3)		
Moderate/severe steatosis	1657 (5.3)	109 (7.6)		

**Notes.**

Sample size, *n* = 32922. Data are expressed as mean SD or median (quartile).

BMI body mass index HDL-C high density lipoprotein cholesterol LDL-C low density lipoprotein cholesterol ALT alamine aminotransferase AST aspartate aminotransferase ALP alkaline phosphatase GGT gamma-glutamyl transferase

### Association between NST changes and NAFLD

**Figure 2:**
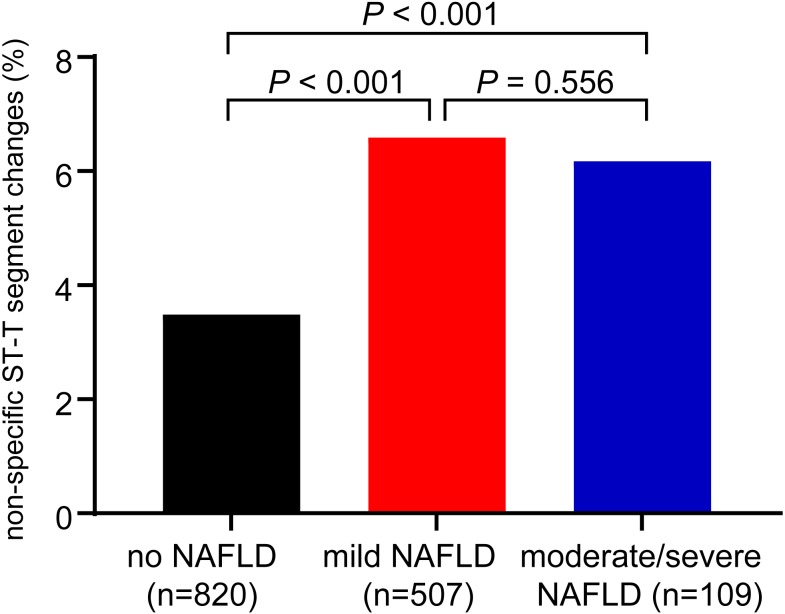
Proportions of non-specific ST-T segment changes in patients with mild NAFLD, moderate/severe NAFLD and without NAFLD. *P* < 0.001 for trend by the chi-squared test.

Remarkably, as shown in Fig. 2, the proportion of the patients with NST changes was associated with the presence of NAFLD on ultrasound (*P* <0.001 for the trend). In patients with no steatosis, mild steatosis and moderate/severe steatosis, the incidence of the NST changes were 3.49%, 6.59% and 6.17%, respectively. However, the proportions of NST changes were not significantly different between patients with mild and moderate/severe steatosis.

As shown in Table 2, the univariate analysis indicated that the association between NAFLD and NST changes was significant, with a 1.925-fold increased OR (95% CI [1.729–2.143]) (model 1). Our data suggested that NAFLD is an independent risk factor for NST changes. Adjustment for age and sex did not detectably weaken the association between NAFLD and NST changes (model 2), the corresponding OR (95% CI) remained 1.935 (95% CI [1.725–2.170]). After further adjustments for other potentially confounding factors including heart rate, hypertension (blood pressure ≥ 140/90 mmHg), FPG, BMI, TC, TG, and HDL-C, NAFLD remained a significant risk factor for NST changes, and the corresponding multivariable-adjusted OR was 1.289 (95% CI [1.122–1.480]). In model 4, we found that age, BMI, hypertension, high FBG, and high TC were also independent predictors of NST changes

**Table 2:** Logistic regression models for NAFLD as a predictor for non-specific ST-T segment changes.

Logistic regression model			OR (95% CI)	*P*-value
**NAFLD (yes vs no)**				
	Model 1		1.925 (1.729–2.143)	<0.001
	Model 2		1.935 (1.725–2.170)	<0.001
	Model 3		1.596 (1.415–1.800)	<0.001
	Model 4		1.289 (1.122–1.480)	<0.001
**Other independent predictors of non-specific ST-T segment changes in Model 4**		
	Sex			
		Female	Reference	
		Male	0.359 (0.317∼0.405)	<0.001
	Age		1.058 (1.052∼1.064)	<0.001
	BMI		1.072 (1.050∼1.093)	<0.001
	Hypertension			
		No	Reference	
		Yes	1.837 (1.622∼2.081)	<0.001
	FPG			
		Normal	Reference	
		High	1.258 (1.104∼1.434)	0.001
	TC			
		Normal	Reference	
		High	1.195 (1.021–1.397)	0.026
	HDL-C			
		Normal	Reference	
		Low	0.839 (0.711–0.989)	0.037

**Notes.**

Sample size, *n* = 32922. Data are expressed as OR (95% CI).

OR odds ratio CI confidence interval BMI body mass index FPG Fasting plasma glucose TC total cholesterol HDL-C high density lipoprotein cholesterol

Covariates included in multivariable regression models were as follows: model 1: unadjusted; model 2: age, sex; model 3: adjustment for heart rate, hypertension (blood pressure ≥140/90 mmHg) and fasting plasma glucose and plus the same variables included in model 2; model 4: adjustment for the same variables included in model 3 plus BMI, triglyceride, total cholesterol, HDL-C.

### Association between NST changes and NAFLD in different subgroups

**Figure 3:**
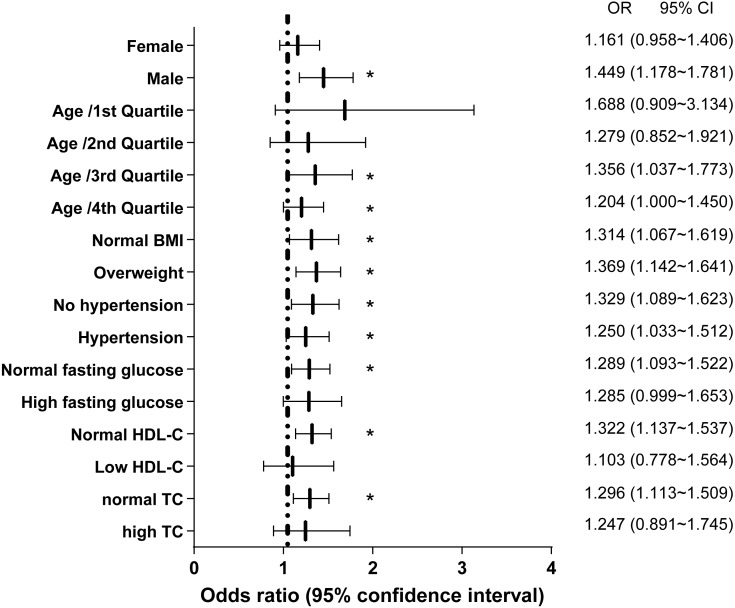
Logistic regression models for NAFLD as a predictor for non-specific ST-T segment changes in different subgroups.

To further elucidate the association between NAFLD and NST changes, we investigated the significance of differences in subgroups stratified by sex, age, BMI, hypertension, fasting glucose, HDL-C, and total cholesterol (Fig. 3). The subgroup analyses indicated that the association between NAFLD and NST changes was stronger in males (OR 1.449, 95% CI: 1.178∼1.781) than in females (OR: 1.161, 95% CI: 0.958∼1.406). Likewise, differences were larger for the 3rd and 4th quartile of age than for the other two quartiles. However, similarly large impacts were observed across participant subgroups for normal BMI or overweight, no hypertension and hypertension. Interesting, it was less relevant in high fasting glucose, low HDL-C and high TC than their respectively references.

## Discussion

In this large-sample retrospective study including records of health examinations, NST changes were significantly associated with NAFLD, notably, the association was independent of potential confounders. In addition, we found that age, sex, hypertension, fasting glucose, TC and HDL-C levels might be significant predictors of NST changes. Our findings suggested that the NST changes might represent an additional correlation with higher CVD risk in patients with NAFLD, and emphasized the importance of evaluating arrhythmic risk in NAFLD patients so as to strengthen early prevention and early intervention of CVD.

Previous studies have demonstrated that there existed significant correlation between NAFLD and cardiac arrhythmias (Ismaiel et al., 2019), such as QTc prolongation (Targher et al., 2014), atrial fibrillation (Anstee et al., 2018), ventricular arrhythmias (Mantovani et al., 2016), but little attention was paid to explore the relationship between NST and NAFLD. Although, it has been suggested that NAFLD have adverse impact on ST segment elevation in myocardial infarction (Emre et al., 2015; Keskin et al., 2017) and NAFLD was correlated with silent myocardial ischemia (Lee et al., 2006), NST changes may be earlier signs of cardiovascular changes (Healy & Lloyd-Jones, 2016). The present study provided evidence indicating that NST changes were significantly associated with NAFLD, which heightened greater vigilance for early prevention of CVD events in patients with NAFLD. However, intriguingly, we found that the incidence of NST in the moderate/severe hepatic steatosis was slightly lower (6.17%) compared to the incidence of NST in patients with mild steatosis (6.59%). We believed the reason was that the population we studied was made up of relatively healthy people under 65 years old undergoing health checkup, and the number of severe steatosis was very few in these patients. We need to further investigate the relationship between NST and NAFLD in the elderly patients and the hospitalized patients.

Consistent with our results, previous studies have reported the risk factors of NST changes, such as age, sex, hypertension, and serum triglyceride levels. The prevalence of NST abnormalities was higher in females (4.5% vs 2.3% in males; *P* <0.001) among healthy volunteers (Hingorani et al., 2012), and similar findings were also observed in an Australian Busselton study (Cullen et al., 1982) and the Michigan study (Ostrander Jr. et al., 1965). One study proposed that NST changes were associated with a remarkably higher incidence rate of unsatisfactory blood pressure control in hypertension patients, especially among diabetic patients (Bao et al., 2017). Kumar et al. also concluded that patients with high blood pressure tended to have NST changes (Kumar et al., 2008). Similarly, an epidemiologic cohort study suggested that the incidence rate of resting ST-T segment abnormalities in men without CHD is greatly affected by age, increasing from 2% at 40 years to 30% at the age of 80 years. Men with such ST-T segment changes were older and had higher serum triglyceride levels and worse glucose tolerance (Sigurdsson et al., 1996). In addition, in line with LDL-C as an independent risk factor for CVD (Moriarty, Gray & Gorby, 2019), our result also substantiated that LDL-C was implicated in NST changes. These previous studies validated our results and further provided insights regarding the potential predictive value of NST changes associated with NAFLD. NST changes associated with NAFLD might reveal an additional connection to increased CVD risk in patients with NAFLD and emphasize the importance of evaluating the cardiovascular and arrhythmic risk for early warning and prevention in those patients.

Although NST changes are significantly associated with NAFLD, the pathophysiological mechanisms have not been fully elucidated. Here, we postulated that the possible mechanisms include the following. First, NST changes might indicate subclinical CVD, early left ventricular hypertrophy, or increased left ventricular mass (Kumar & Lloyd-Jones, 2007). NAFLD might be related to NST due to shared risk factors for cardiac metabolism and co-morbidities or as a marker of coexisting ectopic accumulation of fat in other organs. For example, myocardial steatosis and accumulated pericardial fat might result in myocardial functional and structural disturbances (Fox et al., 2009; Hallsworth et al., 2013). Second, myocardial perfusion is more vulnerable to impairment when the NAFLD score is ≥ 3 (Emre et al., 2015); coincidentally, NST abnormalities were associated with impaired coronary circulation in a healthy population (Larsen et al., 2002). Accumulating evidence suggests that hepatic steatosis may aggravate systemic insulin resistance and alter secretory patterns of hepatokines and proatherogenic factors that may play a role in the pathophysiology of NST changes (Bhatia et al., 2012). Such an association among NST changes, cardiac function and coronary circulation offers a plausible interpretation for the association between NST changes and NAFLD in our study. Despite these assumptions, there is no conclusive data to link NST changes with any specific physiological mechanism in patients with NAFLD. Further study is needed to clarify the pathophysiologic association between NST changes and NAFLD.

There are some limitations of this study. First, this is a cross-sectional study in which the participants were screened based on the inclusion and exclusion criteria, thus leading to difficultly in determining causal relationships between NAFLD and NST changes (Setia, 2016). Second, NAFLD was diagnosed based on US, which is less sensitive when liver fat infiltration is less than approximately 30%. However, this method is potentially a practical tool in clinical practice because of its relatively low cost, non-invasive nature and diagnostic accuracy. Finally, residual confounders were not eliminated due to the measurement errors of these variables. Despite these limitations, our study selected a large sample of healthy individuals who were less likely to be affected by comorbidity-related biases than those in studies conducted in higher risk populations.

## Conclusion

Our study suggested that NST changes were significantly and independently associated with NAFLD after adjusting for potential confounding factors. Further studies are required to investigate the relationship between NST changes and NAFLD in the elderly patients and the hospitalized patients and elucidate the pathophysiological mechanisms and determine the long-term clinical effects of NAFLD on NST changes.

##  Supplemental Information

10.7717/peerj.9090/supp-1 Data S1 Raw data

## List of Abbreviations

NAFLD: Nonalcoholic fatty liver disease
NST: non-specific ST-T segment
ECG: Electrocardiogram
OR: odds ratio
SD: standard deviation
CI: confidence interval
CVD: cardiovascular disease
CHD: coronary heart disease
BP: blood pressures
FPG: fasting plasma glucose
BMI: body mass index
HDL-C: high density lipoprotein cholesterol
TC: total cholesterol
TG: triglyceride
LDL-C: low density lipoprotein cholesterol
ALT: alamine aminotransferase
AST: aspartate aminotransferase
ALP: alkaline phosphatase
GGT: gamma-glutamyl transferase
US: ultrasonography

## Additional Information and Declarations

### Data Availability

The following information was supplied regarding data availability: The raw measurements are available as a Supplemental File.
